# Poster 1025: Introduction to the clinical practice of molecular allergology

**DOI:** 10.1186/1939-4551-7-S1-P15

**Published:** 2014-02-03

**Authors:** Guy Tropper

**Affiliations:** 1 Avant Garde Medical, Boucherville, QC, Canada

## Background

While it has been used clinically in Europe since the early 2000, molecular allergology remains relatively unknown to a number of clinicians involved in the field of allergy and this at a time when the understanding of allergy at the level of proteins allows for novel diagnostic and therapeutic avenues. Molecular allergy offers clinicians a better understanding of the allergy dynamics of certain patients, particularly in some situations of food allergy and in multi-allergy contexts.

## Methods

A introduction to the science of molecular allergology, based on 6 concepts has been developed. A concise yet clinically effective "Molecular allergology summary 2013" card has been devised so as to facilitate the allergy physician's first steps in the clinical use of molecular allergology. Notions of molecular similarities and the relevance of biologic testing results will be reviewed. This will assist initial assessments of specific clinical contexts where molecular allergology will prove particularly useful.

## Results

A coherent appreciation of molecular families, their major component markers and their clinical character adds a whole new dimension to the evaluation and management of patients with various inhalant, nuts and other food allergies. The oral allergy syndrome, from the perspective of the molecular families at play, can be better appreciated for the myriad of syndromes that they really are, each with its own prognostic and therapeutic implications. The pertinence of molecular allergy testing vis-à-vis multi-sensitized respiratory allergy patients, milk and egg allergy management and other more specific clinical conditions can be addressed. Future applications of molecular allergy will likely change considerably some of our therapeutic approaches.

## Conclusions

Molecular allergy can enhance our care of allergy patients today and opens new doors on allergy therapeutics. It also brings new parameters and business-model issues in allergy care in North-America. Beyond the helpfulness of component-resolved diagnosis in the practice of allergy medicine, the leadership of clinical allergists is neeeded in defining its judicious application to the care of our allergic patients.

